# Explainable machine learning model of coronary artery disease combined with diabetes: development and validation study

**DOI:** 10.3389/fcvm.2025.1674287

**Published:** 2026-01-05

**Authors:** Xujie Wang, Shipeng Wang, Xuhui Liu, Rongfei Xie, Shasha Liang, Biyun Wang, Yuke Zhang

**Affiliations:** 1 Department of Emergency ICU, The Affiliated Hospital of Qinghai University, Xining, Qinghai, China; 2 Department of Cardiovascular Medicine, The First Hospital of Jilin University, Changchun, China; 3 Department of Neurology, The Second Hospital of Lanzhou University, Lanzhou, Gansu, China; 4 Department of Biobank, Sun Yat-Sen Memorial Hospital, Sun Yat-Sen University, Guangzhou, China

**Keywords:** coronary artery disease, diabetes mellitus, machine learning, mortality, prognostic model

## Abstract

**Background:**

Coronary artery disease (CAD) demonstrates a strong bidirectional association with diabetes mellitus, which not only elevates cardiovascular disease risk but also correlates with poorer clinical prognosis. Prognostication in patients with comorbid CAD and diabetes remains a critical clinical challenge, significantly influencing therapeutic decision-making. Leveraging readily available clinical parameters for predicting adverse outcomes in this population offers substantial clinical value. This investigation employs machine learning algorithms to develop predictive models for prognostic assessment in CAD patients with diabetes comorbidity.

**Method:**

We conducted a retrospective cohort study of 389 patients with comorbid coronary artery disease (CAD) and diabetes mellitus. The cohort was randomly allocated into a training set (*n* = 273) and an **internal** validation set (*n* = 116). Feature selection utilized LASSO regression followed by backward stepwise Cox regression analysis. A nomogram incorporating independent predictors was developed for clinical application. Model performance was assessed through discrimination metrics, calibration plots, and decision curve analysis (DCA). Random survival forest analysis validated the clinical significance of selected variables.

**Result:**

Our modeling approach employed a systematic methodology: LASSO regression for initial feature selection followed by backward stepwise Cox regression analysis, yielding eight independent predictors.The final model incorporated hemoglobin, INR, albumin, NT-proBNP, age, fibrinogen, diuretic use, and digitalis therapy. The integrated model demonstrated strong discriminative performance for mortality prediction across both training (AUC = 0.846, 0.838, 0.82) and validation cohorts (AUC = 0.824, 0.813, 0.798) at 3-, 5-, and 8-year intervals. Calibration plots and decision curve analysis confirmed model reliability and clinical utility over time. A nomogram was developed to facilitate individualized risk stratification. Kaplan–Meier analysis showed significant survival stratification by individual predictors, and restricted cubic spline analysis identified non-linear associations between continuous variables and mortality. Random survival forest analysis prioritized five key predictors (hemoglobin, INR, albumin, NT-proBNP, age). Comparative evaluation against the 9-variable model confirmed superior performance of the comprehensive model across all timepoints.

**Conclusion:**

Our multimodal prognostic model demonstrated robust performance in predicting all-cause mortality among patients with coronary artery disease and diabetes comorbidity. The nomogram's capacity for personalized risk estimation offers potential utility in clinical decision-making and patient stratification.

## Introduction

The primary cause of death worldwide and a significant contributor to disability is coronary artery disease (CAD) (1). Diabetic patients are more likely to experience severe acute coronary syndromes and have worse outcomes than non-diabetic patients (2, 3). Although microvascular complications substantially impact diabetes-related morbidity, mortality primarily stems from macrovascular manifestations including CAD and cerebrovascular events (4). This clinical landscape underscores the critical need for accurate risk stratification tools to inform therapeutic strategies and healthcare resource utilization.

Conventional prognostic models have predominantly utilized isolated parameters including natriuretic peptide levels, glycated hemoglobin A1c (HbA1c), and New York Heart Association (NYHA) functional classification (5, 6). Established risk assessment tools include the Framingham Risk Score (FRS) in North America and the Systematic Coronary Risk Evaluation (SCORE) model in Europe (7, 8). However, these models demonstrate limited discriminative capacity for CAD mortality prediction and insufficient integration of modern prognostic factors for acute coronary syndrome (9, 10). Furthermore, FRS applicability shows significant variability across ethnic and geographic populations. Most current prognostic models focus on identifying high-risk subgroups rather than providing comprehensive risk stratification. Emerging biomarkers like the triglyceride-glucose (TyG) index show promise for predicting major adverse cardiovascular events (MACE) in post-CABG patients with CAD and diabetes, potentially enabling targeted monitoring and preventive interventions (11). Notably, validated clinical scoring systems for long-term prognosis assessment in comorbid CAD and diabetes populations remain largely unavailable.

The rapid advancement of artificial intelligence has enabled increasing machine learning applications in clinical prediction. Supervised learning algorithms enhance prognostic accuracy by iteratively optimizing model parameters against clinical outcome data (12). This technological evolution creates new opportunities for developing precision prognostic algorithms in diabetes-associated CAD management.

Our multimodal approach comprehensively integrates demographic profiles, clinical histories, pharmacological data, and echocardiographic parameters to enhance risk assessment in comorbid CAD-diabetes patients. We hypothesize this multidimensional strategy will surpass conventional single-parameter models in prognostic precision, ultimately improving clinical risk categorization and therapeutic decision-making.

## Methods

### Study design and participants

Out of 834 patients with CAD and diabetes, 389 met the inclusion and exclusion criteria and were included in the final analysis. This study analyzed clinical data from 389 CAD and diabetes patients treated between December 2014 and December 2024. The primary outcome was cardiovascular death. The study included 389 participants who satisfied the following inclusion criteria and had both diabetes and CAD: (1) aged over 18 years old; (2) diagnosed with CAD according to the European Society of Cardiology (ESC) diagnosis and treatment guidelines (13): a pathological process characterized by atherosclerotic plaque accumulation in the epicardial arteries, whether obstructive or non-obstructive; (3) fasting plasma glucose (FPG) ≥ 7 mmol/L (126 mg/dL) is introduced as the criterion for diagnosis of diabetes (14). Exclusion criteria consisted of (1) Acute or chronic infections are present concurrently; (2) the patient has a tumor, autoimmune illness, or hematological disease; (3) and the patient's information is lacking. Survival outcomes were verified through structured telephone interviews or clinical follow-up documentation.This study adhered to the Declaration of Helsinki and was approved by the Ethics Committee of the Second Hospital of Lanzhou University (2024A-1270), with a waiver of informed consent.

### Data acquisition

At baseline, all participants underwent comprehensive demographic evaluation including sex, age, and clinical histories (hypertension, diabetes mellitus, atrial fibrillation, coronary heart disease, anemia, smoking status, and alcohol use). Medication profiles were documented, encompassing antiplatelet agents, anticoagulants, statins, nitrates, diuretics, and cardiac remodeling therapies. Standardized laboratory assessments included fasting glucose, hemoglobin, lipid profile (total cholesterol, triglycerides, HDL, LDL), renal function (serum creatinine), hepatic enzymes (AST, ALT), albumin, INR (International Normalized Ratio), CRP, and NT-proBNP (N-terminal pro B-type Natriuretic Peptide). Supplementary cardiovascular evaluations comprised 12-lead ECG and echocardiographic parameters.

In the feature selection process, we used LASSO regression to identify significant predictors of patient outcomes. We included time-dependent covariates and interaction terms in the LASSO regression. Conditions like diabetes and hypertension develop over time due to long-term factors. To account for this, we incorporated time-dependent variables to reduce confounding from the chronic nature of these diseases. Additionally, for medication use, we classified patients as “users” if they had been regularly prescribed medications for coronary artery disease and diabetes for six months or more, defining “non-users” as those who did not meet this threshold. This approach allowed us to effectively capture the temporal relationships and reduce potential biases in our analysis. Although time-dependent covariates were considered during variable selection, the final prognostic model was a baseline Cox proportional hazards model. Medication exposure variables (e.g., diuretics and digoxin) were constructed using prescription records from the first six months after the index date; patients who continuously received these medications for at least six months were classified as “users”, whereas those who did not meet this threshold were classified as “non-users”. Consequently, a formal time-dependent Cox model was not fitted and only baseline covariates were used in the final analysis.

**Figure 1:**
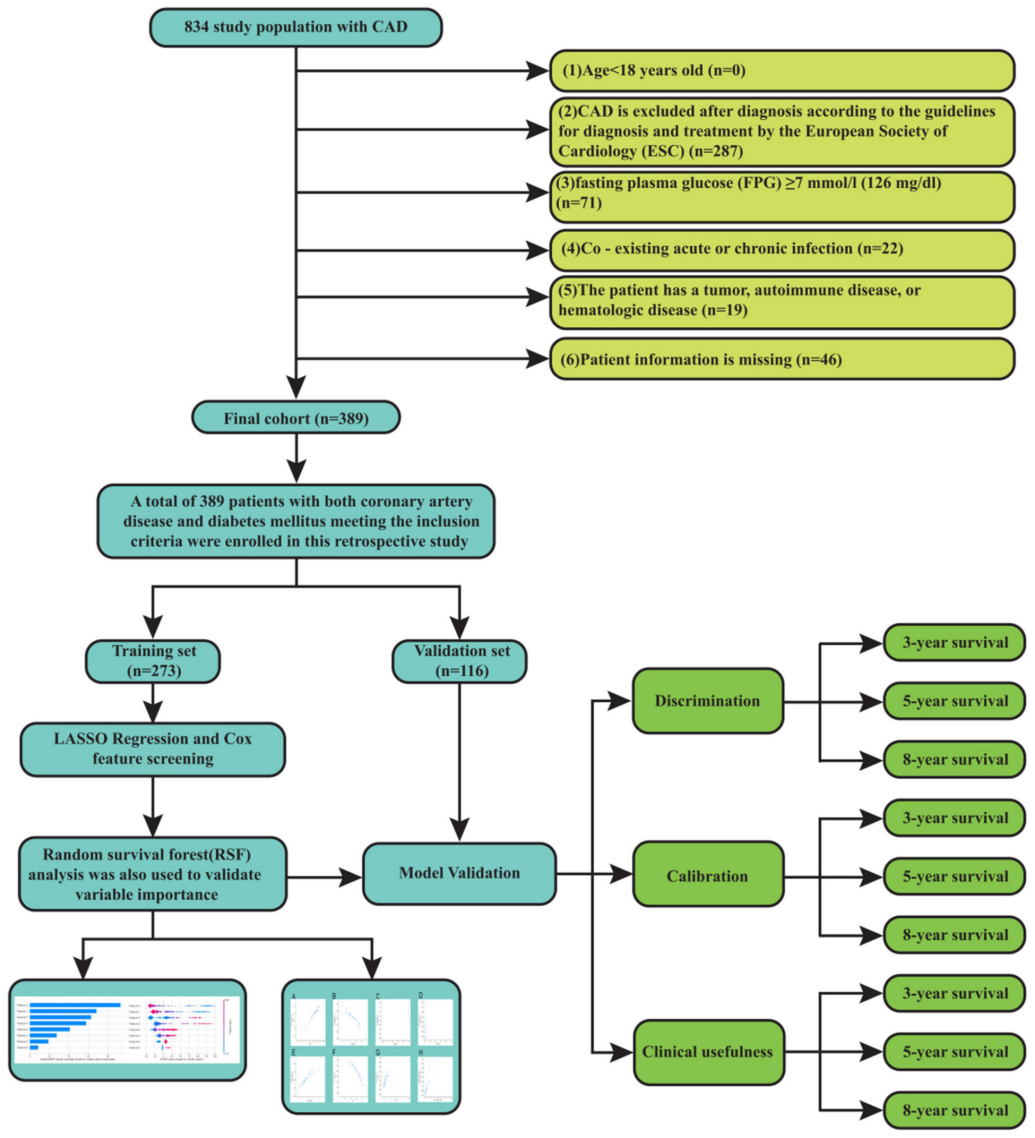
Flow chart.

The final cohort comprised 389 patients with confirmed CAD and diabetes. The training set was defined as 116 patients, and their early morning fasting peripheral blood samples were retained for the test of laboratory examination results. The training set consisted of 273 patients, as shown in Figure 1.

### DStatistical analysis and model development

All statistical analyses and model construction were done using R software (version 4.3.1). The survival and rms packages were used for Cox proportional hazards modelling, random ForestSRC was used to construct the random survival forest, time-ROC and risk Regression were used to estimate time-dependent ROC curves and AUCs, rms and pec were used to generate calibration curves, and dcurves and rmda were used for decision-curve analysis. A summary of functions and packages is provided in Supplementary Table S7. Group comparisons employed *χ*^2^ tests for categorical variables and Mann–Whitney *U*-tests for nonparametric continuous data. Statistical significance was determined using two-tailed α = 0.05. for the handling of missing data and continuous predictors, continuous variables with a missing rate of less than 5% were imputed using the random forest method. Feature selection followed a two-step process: LASSO regression with 10-fold cross-validation was first applied to the training cohort to identify potential predictors, followed by backward stepwise Cox regression. The variance inflation factor (VIF) was used to assess multicollinearity among selected variable. Several methods were used to assess the model's performance. R software (version 4.3.1; R Foundation for Statistical Computing, Vienna, Austria) was used for all statistical analyses. Categorical variables were compared using *χ*^2^ tests, and continuous variables with non-normal distribution were compared using the Mann–Whitney *U*-test. A two-tailed alpha of 0.05 was considered statistically significant. Feature selection followed a two-step process: first, LASSO regression with 10-fold cross-validation was applied to identify predictors. These variables chosen by LASSO were then subjected to backward stepwise Cox regression analysis. The variance inflation factor (VIF) was used to evaluate multicollinearity among the final variables that were chosen. Several methods were used to assess the model's performance. Time-dependent receiver operating characteristic (ROC) curves for 3-, 5-, and 8-year mortality forecasts in both training and validation sets were used to evaluate discrimination. These time horizons were selected to reflect short−, intermediate− and long−term follow−up. The median follow−up (survival) time in our cohort was 4.68 years (interquartile range 0.55–8.71 years), so 5 years approximated the mid-point of follow−up and 8 years corresponded to the upper quartile. As shown in Supplementary Figure S6, follow−up durations ranged from less than 1 year to more than 10 years, with a small peak of patients completing long−term follow−up around 8 years. At 5 years, 184 participants were still under observation, and 111 participants remained at risk at 8 years (Supplementary Figure S6). Evaluating the model at 3, 5 and 8 years therefore captures short−, medium− and longer−term risk while maintaining adequate numbers at risk for reliable estimates. To evaluate the degree of agreement between expected and observed results, calibration curves were plotted. Decision curve analysis (DCA) was used to assess the model's clinical utility. Using Kaplan–Meier survival analyses, the prognostic value of categorical factors was evaluated. The “survminer” package was used to find the best cutoff values for continuous variables. Using (restricted cubic splines) RCS with three knots positioned at the 10th, 50th, and 90th percentiles to evaluate possible non-linear connections, the relationships between continuous variables and mortality were investigated. To offer a useful tool for predicting personal risk, a nomogram was created. The top 8 most significant factors from our 9 predictors were determined by additional analysis using a random survival forest with 1,000 trees. Iterative testing from 1 to 75 was used to establish the ideal node size. We next performed comparative studies between models built utilizing these top 8 variables and our 9-variable model. Time-dependent ROC curves, calibration plots, and decision curve analyses at 3, 5, and 8-year time points in both training and validation cohorts were used to compare the models. Internal validation was performed via bootstrap resampling: we estimated optimism−corrected concordance index (C−index) values and computed 95% bias−corrected and accelerated (BCa) confidence intervals from the bootstrap resamples.

## Results

### Patient characteristics and outcomes

The baseline clinical characteristics, treatment of the total cohort (*N* = 389) are detailed in Supplementary Table S1. Of the 389 patients with CAD combined with diabetes, the median age was 86.00 [82.00;90.00] years, including 299 (76.86%) males and 90 (23.14%) females. The median body mass index (BMI) was 24.46 [22.32;26.75] kg/m^2^. The most common comorbidities were hypertension (341, 87.66%), anemia (172, 44.22%), chronic heart failure (127, 32.65%), and chronic kidney disease (121, 31.11%). In terms of laboratory tests, the median NT-proBNP level was 0.5 × 10^3^ pg/mL (IQR: 0.18–1.91), and the median HDL level was 1.01 mmol/L (0.84; 1.19). Baseline characteristics are shown in Table 1. The cohort was randomly allocated into a training set (*n* = 273) and **an internal validation set (*n* = 116)**. No substantial differences were observed between the training group and the validation group across the majority of features, it indicates that the baseline characteristics of the training cohort and the validation cohort are generally comparable. Supplementary Figure S6 illustrates the distribution of follow-up durations and the number of participants at risk: follow-up durations ranged from less than 1 year to more than 10 years, with a median follow-up time of 4.68 years; 184 participants remained under follow-up at 5 years, and 111 participants remained at risk at 8 years.

**Table 1:** Baseline characteristics of the training and validation groups.

Characteristics	ALL *N* = 389	Validation group *N* = 116	Training group *N* = 273	*P* value
Death				0.336
No	90 (23.14%)	31 (26.72%)	59 (21.61%)	
Yes	299 (76.86%)	85 (73.28%)	214 (78.39%)	
Survival time (year)	4.68 [0.55;8.71]	5.51 [0.81;8.77]	3.77 [0.50;8.23]	0.233
Gender				1.000
Female	45 (11.57%)	13 (11.21%)	32 (11.72%)	
Male	344 (88.43%)	103 (88.79%)	241 (88.28%)	
Age	86.00 [82.00;90.00]	86.00 [84.00;89.00]	87.00 [82.00;90.00]	0.941
BMI (kg/m^2^)	24.46 [22.32;26.75]	23.88 [21.73;26.33]	24.62 [22.53;27.05]	0.077
HR (bpm)	72.00 [65.00;80.00]	72.00 [65.00;80.00]	72.00 [65.00;80.00]	0.996
mSBP (mmHg)	135.40 (13.41)	132.98 (12.51)	136.43 (13.66)	0.016
mDBP (mmHg)	68.60 [62.80;73.60]	68.75 [61.60;73.23]	68.60 [63.33;74.00]	0.335
Smoke				0.364
Never/ Former smoker	243 (62.47%)	68 (58.62%)	175 (64.10%)	
Current smoker	146 (37.53%)	48 (41.38%)	98 (35.90%)	
Hypertension				0.950
No	48 (12.34%)	15 (12.93%)	33 (12.09%)	
Yes	341 (87.66%)	101 (87.07%)	240 (87.91%)	
CHD				0.889
No	268 (68.89%)	81 (69.83%)	187 (68.50%)	
Yes	121 (31.11%)	35 (30.17%)	86 (31.50%)	
CHF				0.426
No	262 (67.35%)	82 (70.69%)	180 (65.93%)	
Yes	127 (32.65%)	34 (29.31%)	93 (34.07%)	
CKD				1.000
No	240 (61.70%)	72 (62.07%)	168 (61.54%)	
Yes	149 (38.30%)	44 (37.93%)	105 (38.46%)	
AF				0.993
No	317 (81.49%)	94 (81.03%)	223 (81.68%)	
Yes	72 (18.51%)	22 (18.97%)	50 (18.32%)	
Anemia				0.533
No	217 (55.78%)	68 (58.62%)	149 (54.58%)	
Yes	172 (44.22%)	48 (41.38%)	124 (45.42%)	
Glucose	5.80 [4.98;7.18]	5.53 [4.90;6.69]	5.84 [5.12;7.40]	0.053
NT_proBNP_1,000 (pg/mL/1,000)	0.50 [0.18;1.91]	0.32 [0.14;1.77]	0.56 [0.20;2.00]	0.062
Hb (g/L)	122.00 [107.00;134.00]	122.00 [106.75;134.00]	121.00 [107.00;134.00]	0.690
TC (mmol/L)	3.65 [3.16;4.28]	3.59 [3.06;4.34]	3.69 [3.20;4.28]	0.496
TG (mmol/L)	1.32 [0.99;1.92]	1.32 [0.92;1.97]	1.32 [1.01;1.82]	0.898
HDL (mmol/L)	1.01 [0.84;1.19]	1.02 [0.82;1.23]	1.01 [0.85;1.18]	0.914
LDL (mmol/L)	2.03 [1.54;2.52]	2.01 [1.58;2.52]	2.03 [1.51;2.51]	0.994
Albumin (g/L)	38.50 [35.00;40.60]	38.50 [35.88;41.32]	38.40 [35.00;40.50]	0.220
Scr (μmol/L)	90.80 [72.00;123.00]	89.00 [71.25;126.88]	91.10 [72.10;122.70]	0.960
UA (μmol/L)	330.50 [237.60;420.00]	336.55 [235.92;418.52]	328.60 [241.20;420.00]	0.846
Na (mmol/L)	139.00 [136.00;141.00]	139.50 [136.00;142.00]	139.00 [136.00;141.00]	0.191
K (mmol/L)	4.00 [3.78;4.31]	4.00 [3.75;4.38]	4.00 [3.79;4.31]	0.898
Ca (mmol/L)	2.23 [2.13;2.32]	2.24 [2.14;2.34]	2.22 [2.12;2.31]	0.211
*P* (mmol/L)	1.10 [0.96;1.25]	1.10 [0.94;1.21]	1.10 [0.97;1.27]	0.402
NLratio	2.48 [1.80;4.09]	2.40 [1.76;4.09]	2.62 [1.80;4.04]	0.457
Fibrinogen (g/L)	3.49 [2.93;4.22]	3.37 [2.87;4.18]	3.57 [2.97;4.23]	0.218
INR	1.08 [1.01;1.15]	1.08 [1.02;1.13]	1.08 [1.01;1.16]	0.904
CRP (mg/L)	0.68 [0.18;2.73]	0.46 [0.12;3.11]	0.74 [0.22;2.70]	0.212
eGFR (mL/min/1.73 m^2^)	67.35 (24.40)	66.67 (23.81)	67.64 (24.68)	0.716
IVS (mm)	11.00 [10.00;12.00]	11.00 [10.00;12.00]	11.00 [10.00;11.00]	0.352
LVPW (mm)	10.00 [10.00;11.00]	10.00 [10.00;11.00]	10.00 [10.00;11.00]	0.948
LVESD (mm)	33.00 [31.00;35.00]	33.00 [31.00;35.00]	33.00 [31.00;35.00]	0.975
LVEDD (mm)	49.00 [47.00;51.00]	49.00 [46.00;51.00]	49.00 [47.00;51.00]	0.425
LVEF (%)	59.00 [55.00;62.00]	59.00 [55.75;62.00]	59.00 [54.00;62.00]	0.595
LVMI (g/m^2^)	32.00 [29.00;34.00]	32.00 [29.75;34.00]	32.00 [29.00;33.00]	0.544
Aspirin				0.916
No	208 (53.47%)	63 (54.31%)	145 (53.11%)	
Yes	181 (46.53%)	53 (45.69%)	128 (46.89%)	
Clopidogrel				0.430
No	144 (37.02%)	39 (33.62%)	105 (38.46%)	
Yes	245 (62.98%)	77 (66.38%)	168 (61.54%)	
β-blocker				0.825
No	106 (27.25%)	33 (28.45%)	73 (26.74%)	
Yes	283 (72.75%)	83 (71.55%)	200 (73.26%)	
CCB				0.695
No	111 (28.53%)	31 (26.72%)	80 (29.30%)	
Yes	278 (71.47%)	85 (73.28%)	193 (70.70%)	
Nitrate				0.391
No	50 (12.85%)	18 (15.52%)	32 (11.72%)	
Yes	339 (87.15%)	98 (84.48%)	241 (88.28%)	
ACEI/ARB				0.931
No	174 (44.73%)	51 (43.97%)	123 (45.05%)	
Yes	215 (55.27%)	65 (56.03%)	150 (54.95%)	
Statins				0.808
No	129 (33.16%)	40 (34.48%)	89 (32.60%)	
Yes	260 (66.84%)	76 (65.52%)	184 (67.40%)	
Diuretics				0.738
No	151 (38.82%)	47 (40.52%)	104 (38.10%)	
Yes	238 (61.18%)	69 (59.48%)	169 (61.90%)	
Digitalis				0.180
No	318 (81.75%)	100 (86.21%)	218 (79.85%)	
Yes	71 (18.25%)	16 (13.79%)	55 (20.15%)	

### Prediction model built based on lasso-cox regression

**Figure 2:**
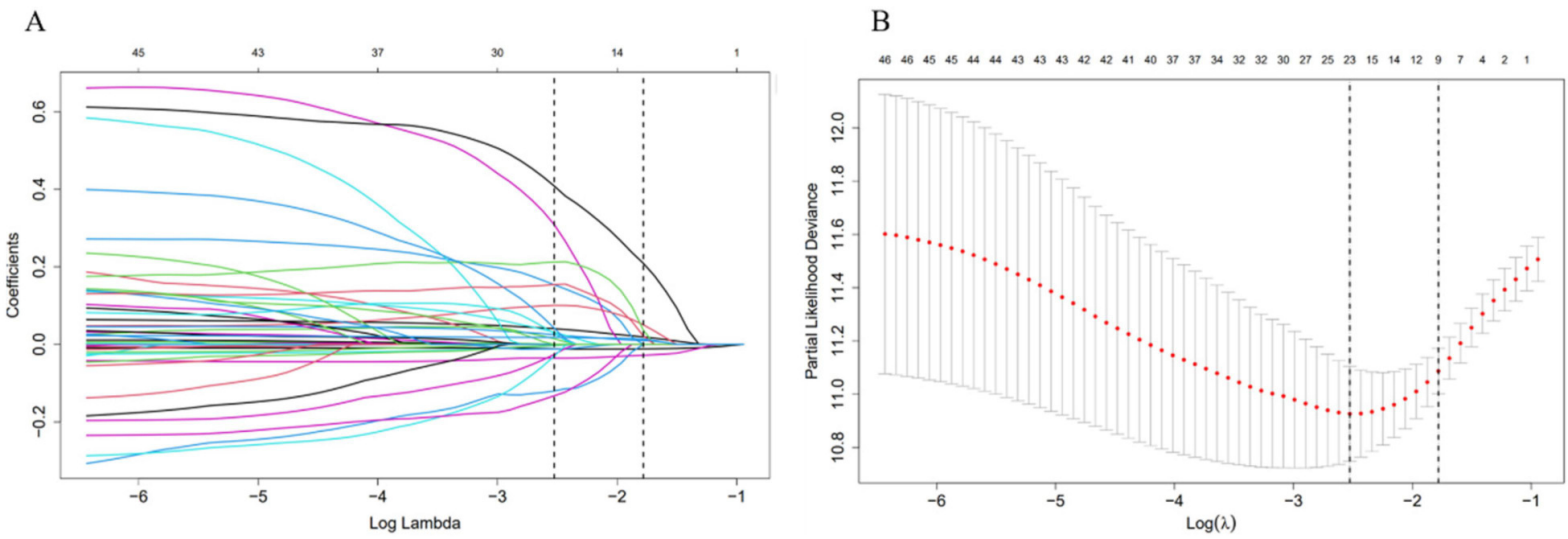
Feature selection using LASSO regression. **(A)** The variation characteristics of the coefficient of variables; **(B)** the selection process of the optimum value of the parameter *λ* in the Lasso regression model by cross-validation method.

Parameters were screened using Lasso regression, and Figure 2A displayed the variation features of the variables' coefficients. After applying the 10-fold cross-validation approach to the iterative analysis, a model with a low number of variables and outstanding performance was achieved when *λ* was 0.063 (Log *λ* = −1.20) (Figure 2B). Age, CRP, Hb, ALB, INR, NT-proBNP, fibrinogen, digitalis, and diuretics were among the factors that were screened. Lasso regression-screened parameters were used to further develop the Cox regression model (Table 2). Detailed coefficients of all selected variables are presented in Supplementary Table S2. Variance inflation factor analysis confirmed the absence of significant multicollinearity among these predictors (Supplementary Table S3).

**Table 2:** Cox proportional hazards regression to predict prognosis based on Lasso regression.

Characteristics	B	SE	HR	CI	Z	*P*
Age	0.054	0.012	1.056	1.031–1.081	4.483	0
Hb	−0.015	0.004	0.985	0.977–0.993	−3.525	0
Albumin	−0.044	0.021	0.957	0.918–0.998	−2.053	0.04
Fibrinogen	0.201	0.064	1.222	1.079–1.385	3.146	0.002
INR	0.574	0.183	1.775	1.241–2.539	3.14	0.002
Diuretics	0.559	0.163	1.749	1.27–2.408	3.423	0.001
Digitalis	0.406	0.195	1.501	1.024–2.201	2.082	0.037
NT_proBNP_1,000	0.027	0.015	1.027	0.997–1.058	1.762	0.078

### The predictive accuracy of the new prognostic model

**Figure 3:**
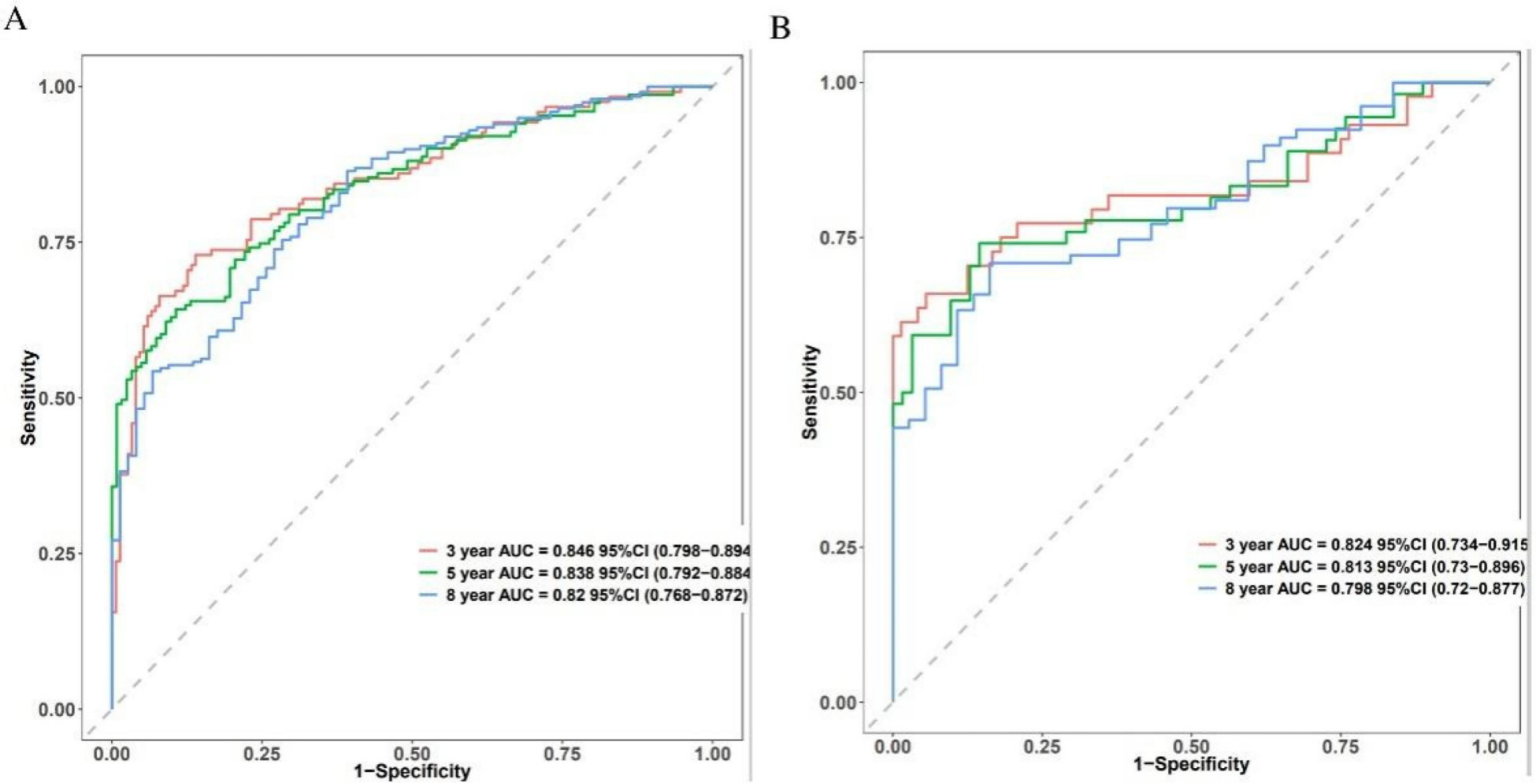
The predictive accuracy and discrimination of the novel prognostic model. Time-dependent ROC in the training cohort **(A)** and validation cohort **(B)**.

Time-dependent receiver operating characteristic (ROC) curves for 3-, 5-, and 8-year death forecasts were used to assess the model's discriminative ability (Figure 3). These results were confirmed in the validation cohort, with AUCs of 0.824 (95% CI: 0.734–0.915), 0.813 (95% CI: 0.73–0.896), and 0.798 (95% CI: 0.72–0.877) for 3-, 5-, and 8-year mortality (Figure 3B). The training set's C-index was 0.753 (95% CI: 0.722–0.784), and the validation set's was 0.74 (95% CI: 0.685–0.795). These C-index estimates and 95% confidence intervals are optimism-corrected values derived using BCa bootstrap resampling; the validation cohort therefore represents an internal validation set.

### Model calibration assessment

**Figure 4:**
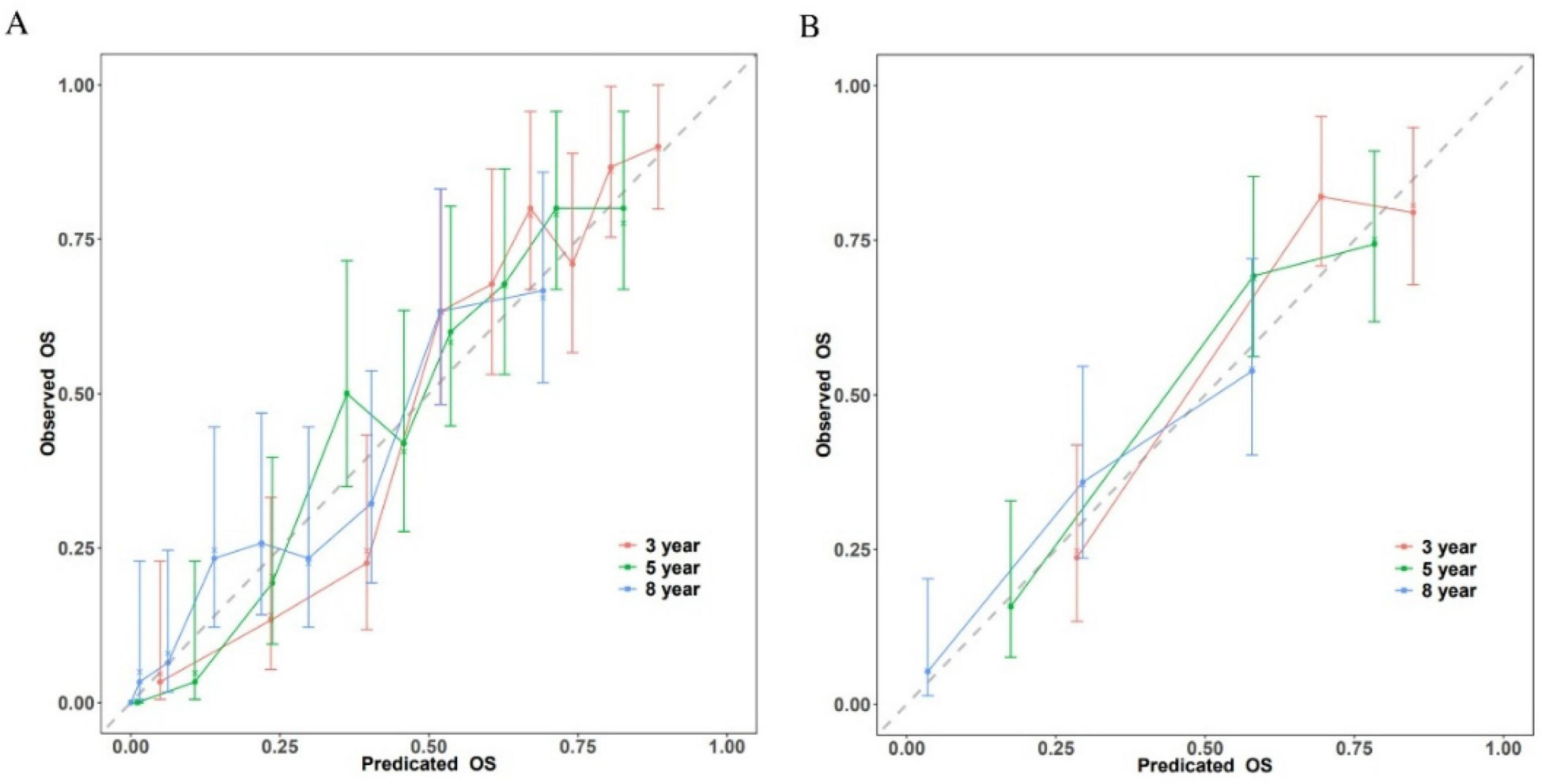
Calibration plots of the nomogram for 3-, 5-, and 8-year survival prediction. **(A)** Training cohort. **(B)** Validation cohort.

Calibration plots comparing expected and observed survival probabilities at 3-, 5-, and 8-year time intervals were used to assess the model's calibration (Figure 4). All three time points’ calibration curves nearly matched the 45-degree diagonal line, showing that the anticipated probability and the actual observations agreed well.

### Clinical decision curve analysis

**Figure 5:**
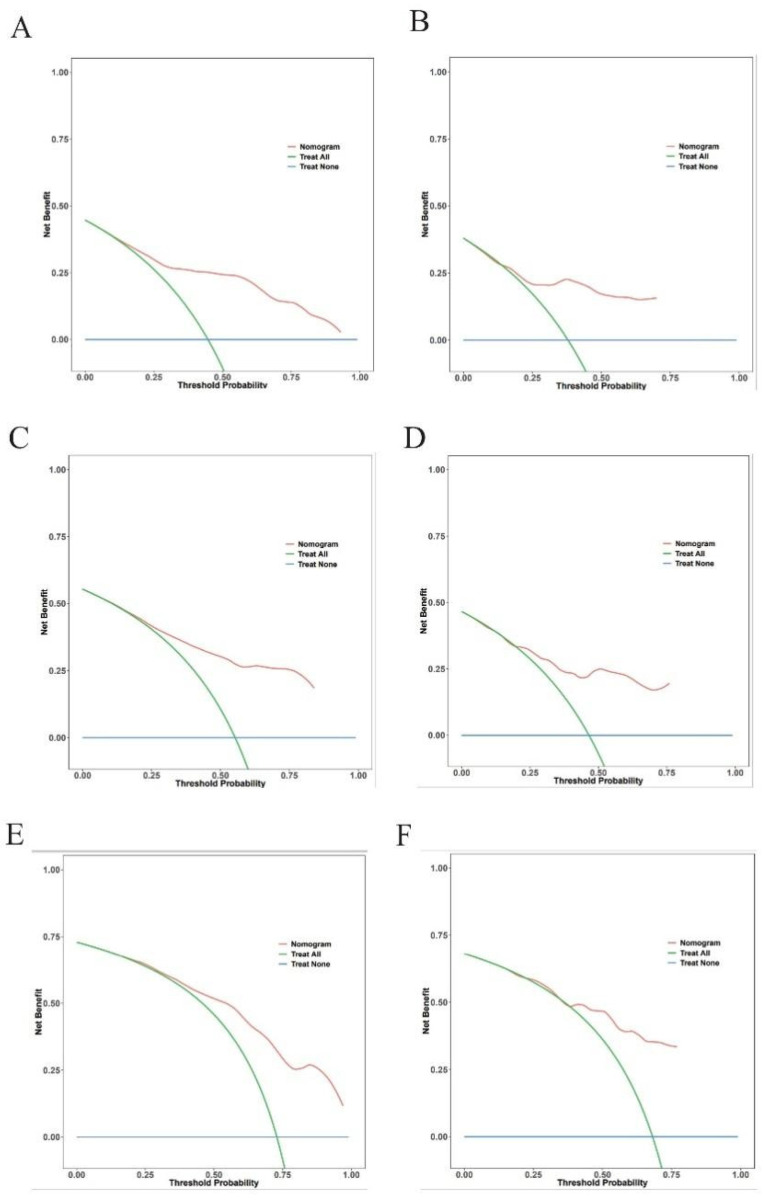
Decision curve analysis for the prediction model. Decision curves for 3-year **(A,B)**, 5-year **(C,D)**, and 8-year **(E,F)**.

Within a reasonable range of threshold probabilities (roughly 0.1 to 0.6), the model outperformed both “treat all” and “treat none” strategies in the training cohort, demonstrating consistent net benefit across a range of threshold probabilities at 3-year (Figure 5A), 5-year (Figure 5C), and 8-year (Figure 5E) follow-up. At the 3-year (Figure 5B), 5-year (Figure 5D), and 8-year (Figure 5F) time points, the validation cohort showed comparable patterns of net benefit, indicating its possible therapeutic utility in directing treatment choices.

### Construction of a prediction nomogram in training cohort

**Figure 6:**
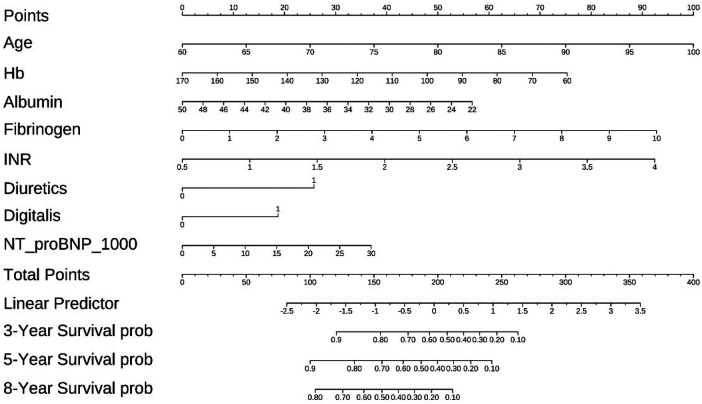
The nomogram for predicting overall survival of coronary artery disease combined with diabetes patients.

A nomogram for predicting 3-, 5-year and 8-years overall survival of coronary artery disease combined with diabetes patients that integrated the above 8 independent prognostic factors is shown in Figure 6. The sum of the points allotted to each predictor—which ranged from 0 to 400 points—was used to determine the total points. The nomogram score of age ranged from 60 to 100, making the largest contribution to the prognosis of coronary artery disease combined with diabetes.

### Kaplan–Meier analysis

**Figure 7:**
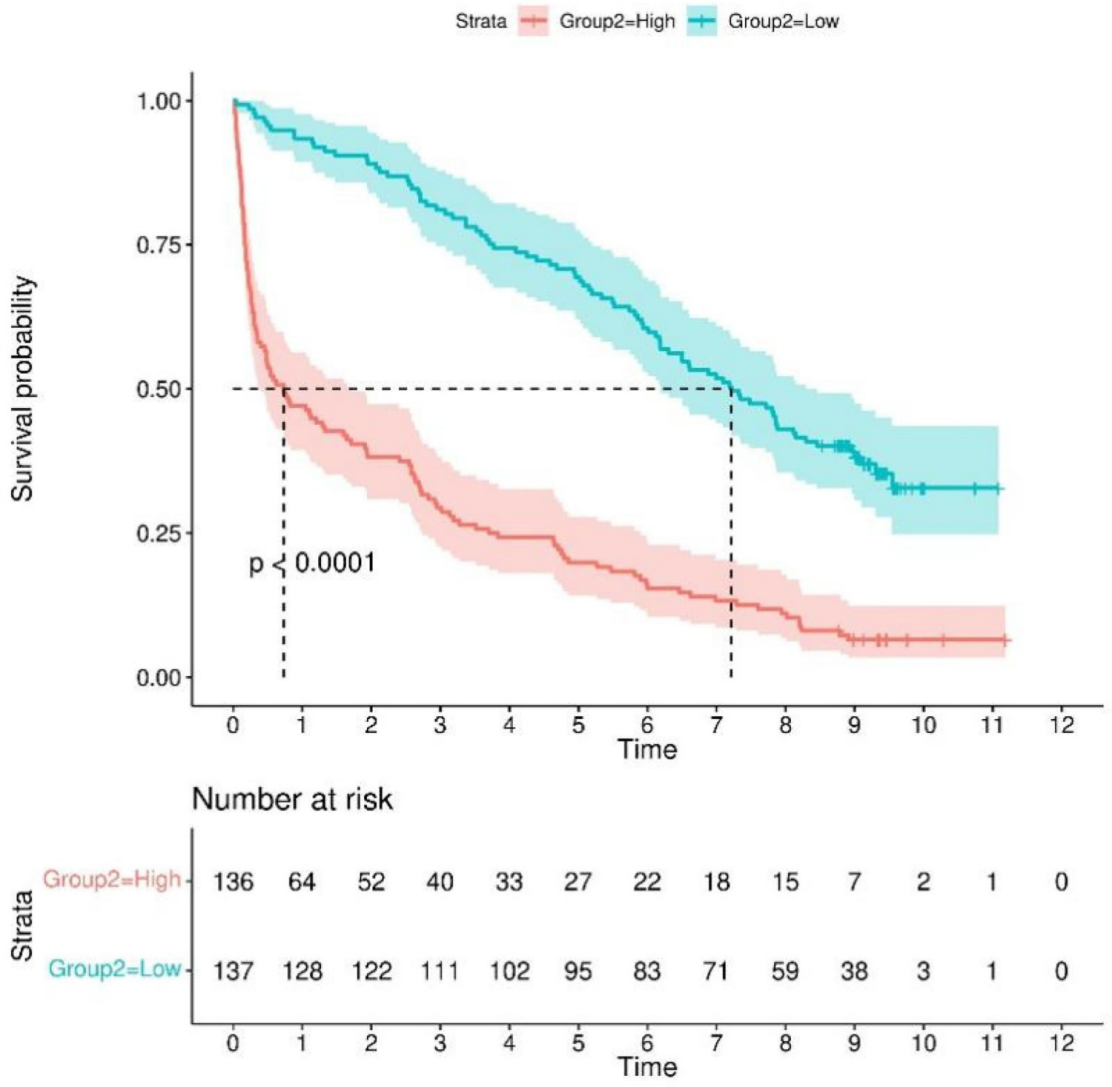
Kaplan–meier survival curves for continuous variables in the final model.

In the Kaplan–Meier analysis, the model displayed good capability in coronary artery disease combined with diabetes patients into high-risk and low-risk groups (Log-rank *P* < 0.0001) in the training set (Figure 7).

### Random survival forest analysis

**Figure 8:**
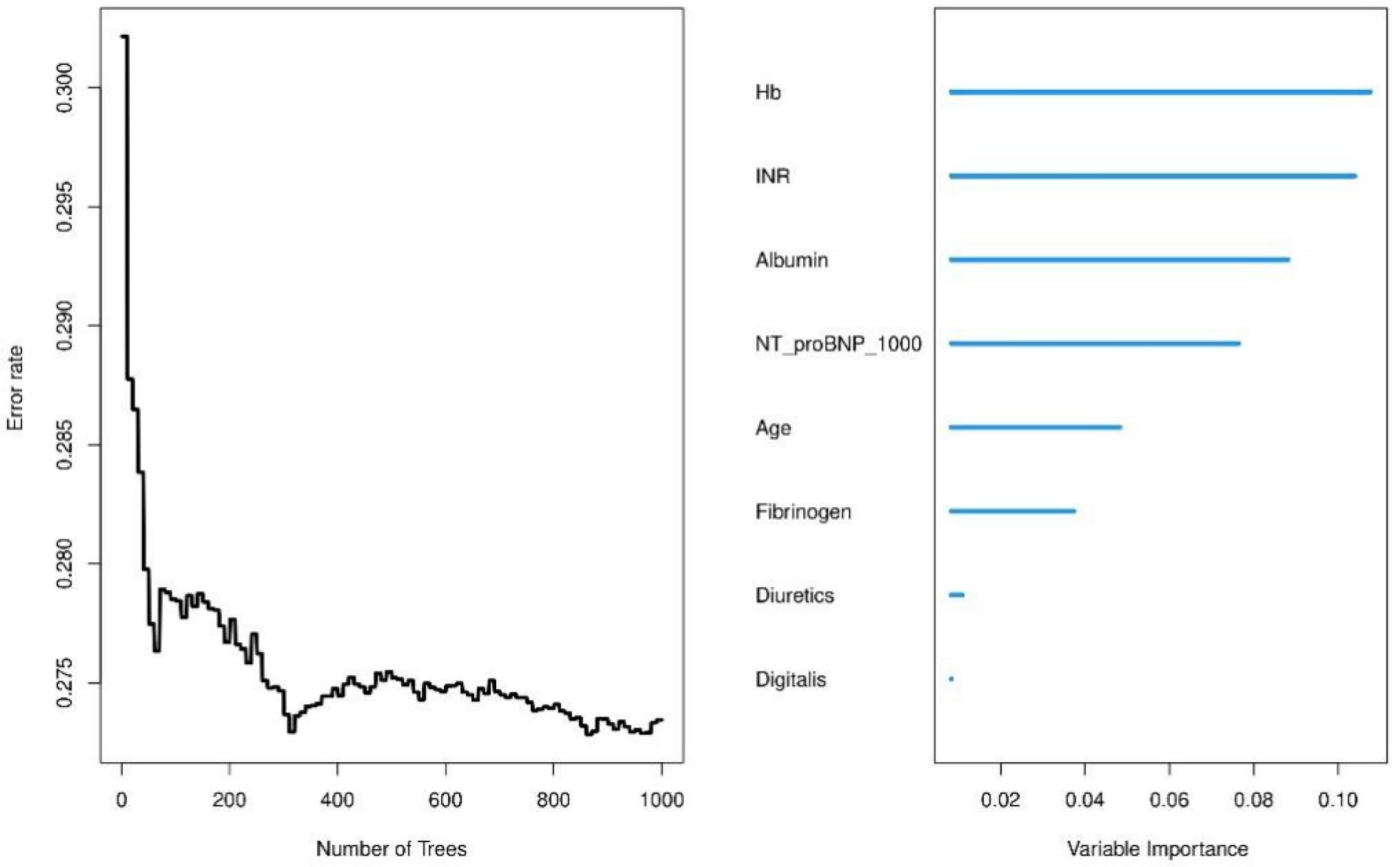
Variable importance analysis from random survival forest model.

We use a training cohort to construct a random survival forest model in order to evaluate the significance of the variables and compare them with the Cox model. Hemoglobin and INR were the best predictors, according to variable importance analysis, followed by albumin, NT-proBNP, patient age, fibrinogen, diuretics and digitalis (Figure 8). After roughly 400 trees, the error rate tends to settle, suggesting that the model has sufficiently converged. In our analysis, we employed Random Survival Forest (RSF) to assess the contribution of each feature to predicting patient outcomes. Additionally, C-index values were compared for both the training and test datasets for both models (Supplementary Figure S3A). The results revealed comparable discriminatory performance, with RSF showing similar or slightly improved performance in both training and test sets. To further evaluate model performance, we compared the Brier scores of Random Survival Forest (RSF) and Cox proportional hazards (CoxPH) models, which are shown in Supplementary Figure S3B. The Brier score comparison demonstrated that RSF had slightly better calibration than CoxPH. To ensure the transparency and interpretability of the model, we utilized SHAP (Shapley Additive Explanations) values, which allow us to explain how each feature influences the model's output. Specifically, we used SHAP summary bar plots (Supplementary Figure S4A) and beeswarm plots (Supplementary Figure S4B) to visualize feature importance. Additionally, we generated SHAP partial dependence plots (Supplementary Figure S5) to explore the non-linear relationships between continuous features and the model's predictions. These plots provide insight into how variations in feature values affect model output, capturing the complex interactions that are often overlooked in traditional linear models. Hyperparameter settings of the random forest are provided in Supplementary Table S5.

### Model comparison across different time horizons

**Figure 9:**
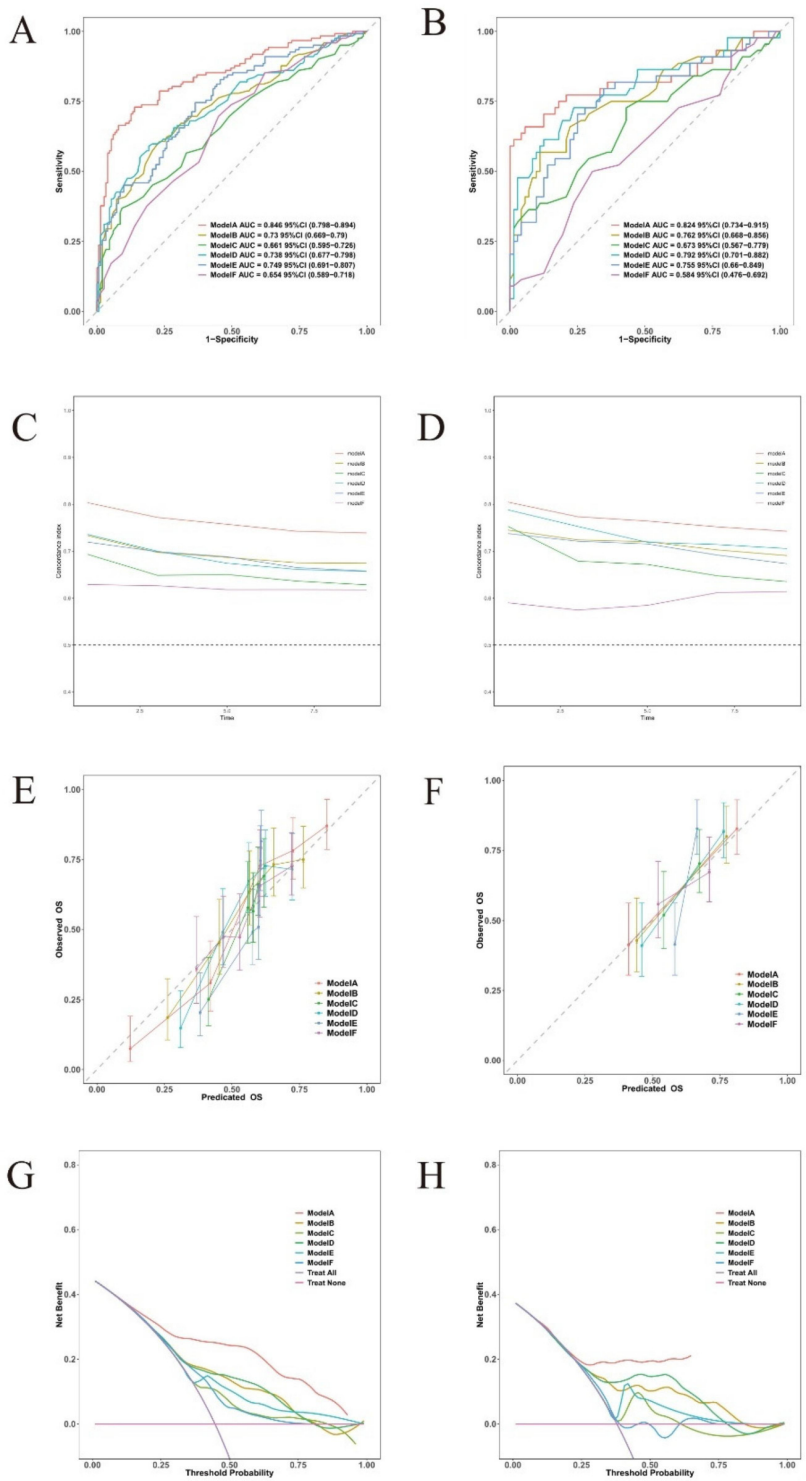
Discriminative performance of different models for 3-year mortality prediction. Model **A**: age + hemoglobin + albumin + fibrinogen + INR + diuretics + digoxin + NT-ProBNP/1,000, Model **B**: hemoglobin, Model **C**: INR, Model **D**: albumin, Model **E**: NT-ProBNP/1,000, and Model **F**: age. **(A,B)** time-dependent ROC curves; **(C,D)** C-index over time; **(E,F)** calibration plots; **(G,H)** decision curve analysis; **(A,C,E,G)** training cohort; **(B,D,F,H)** validation cohort.

We conducted a comprehensive comparison of mortality prediction models across 3-, 5-, and 8-year intervals (Figure 9). In the training cohort, Model A (incorporating all 8 variables) demonstrated superior discriminative performance for 3-year mortality prediction compared to single-variable models (AUC = 0.846, 95% CI: 0.798–0.894). This advantage persisted for 5-year (AUC = 0.838, 95% CI: 0.792–0.884) and 8-year predictions (AUC = 0.82, 95% CI: 0.768–0.872). Model A maintained consistent performance in the validation cohort across all time points: 3-year AUC = 0.824 (95% CI: 0.734–0.915), 5-year AUC = 0.813 (95% CI: 0.73–0.896), and 8-year AUC = 0.798 (95% CI: 0.72–0.877). (Supplementary Figures S1, S2) Calibration plots demonstrated strong agreement between predicted and observed probabilities at all time intervals. Decision curve analysis consistently showed that Model A provided superior net benefit across a wider range of threshold probabilities compared to both simplified models and default strategies.

### Survival analysis based on key predictors

**Figure 10:**
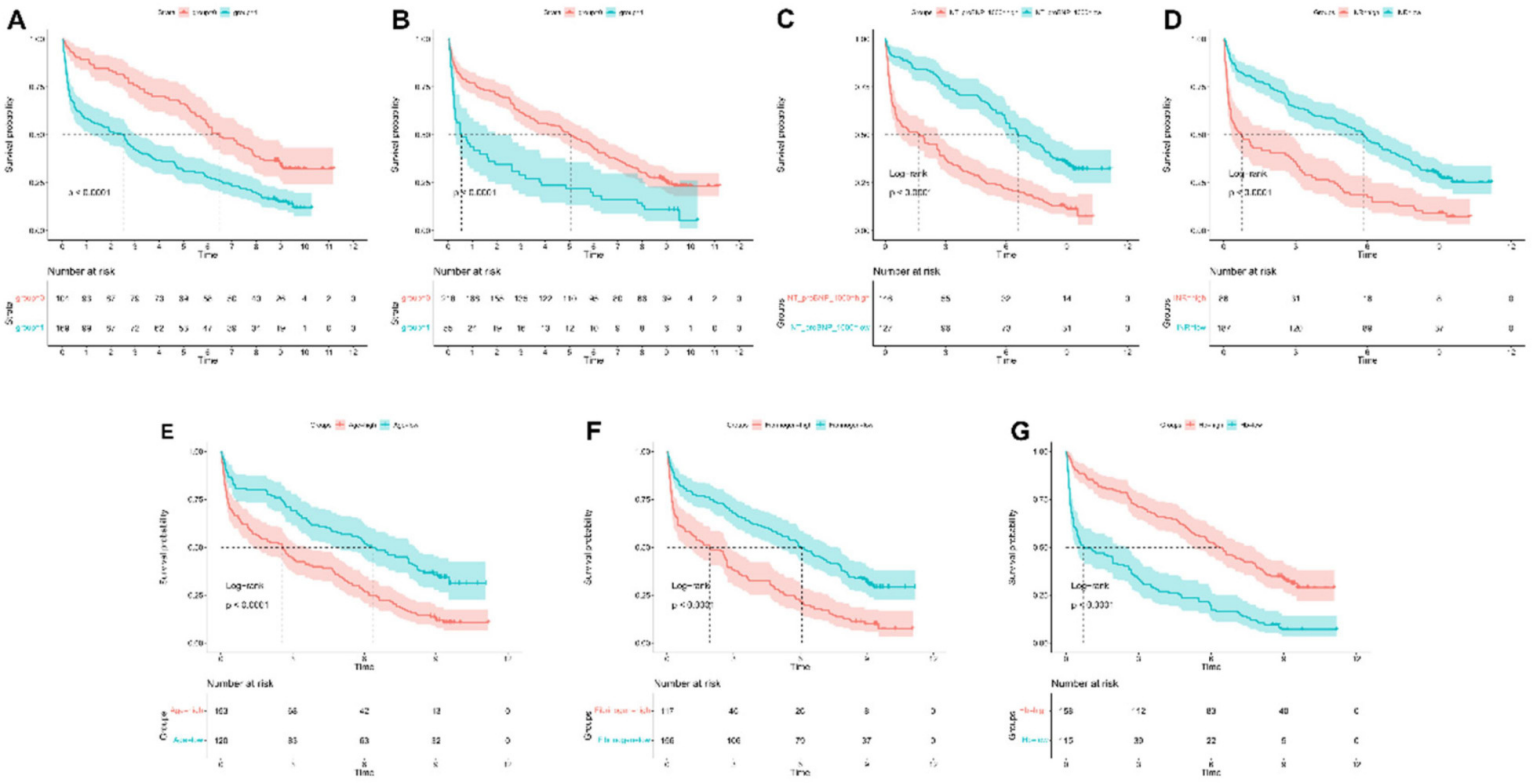
Kaplan–meier survival curves for continuous variables in the final model. Kaplan-Meier survival curves stratified by **(A)** NT-proBNP, **(B)** INR, **(C)** age, **(D)** fibrinogen, **(E)** hemoglobin, **(F)** diuretic use, and **(G)** digoxin use.

Kaplan–Meier analysis was used to evaluate survival differences, with stratification according to key predictors identified in the nomogram analysis (Figure 10). For continuous variables, optimal cutoff values were determined using statistical methods to maximize separation between risk groups (Supplementary Table S4). NT-proBNP (cutoff: 487.2 pg/mL), INR (cutoff: 1.12), age (cutoff: 85 years), fibrinogen (cutoff: 3.72 g/L), and hemoglobin (cutoff: 116 g/L) all demonstrated significant survival outcome stratification (*P* < 0.0001 for all). For categorical variables, the use of diuretics and digoxin also showed strong prognostic stratification (*P* < 0.0001).

### Nonlinear association analysis

**Figure 11:**
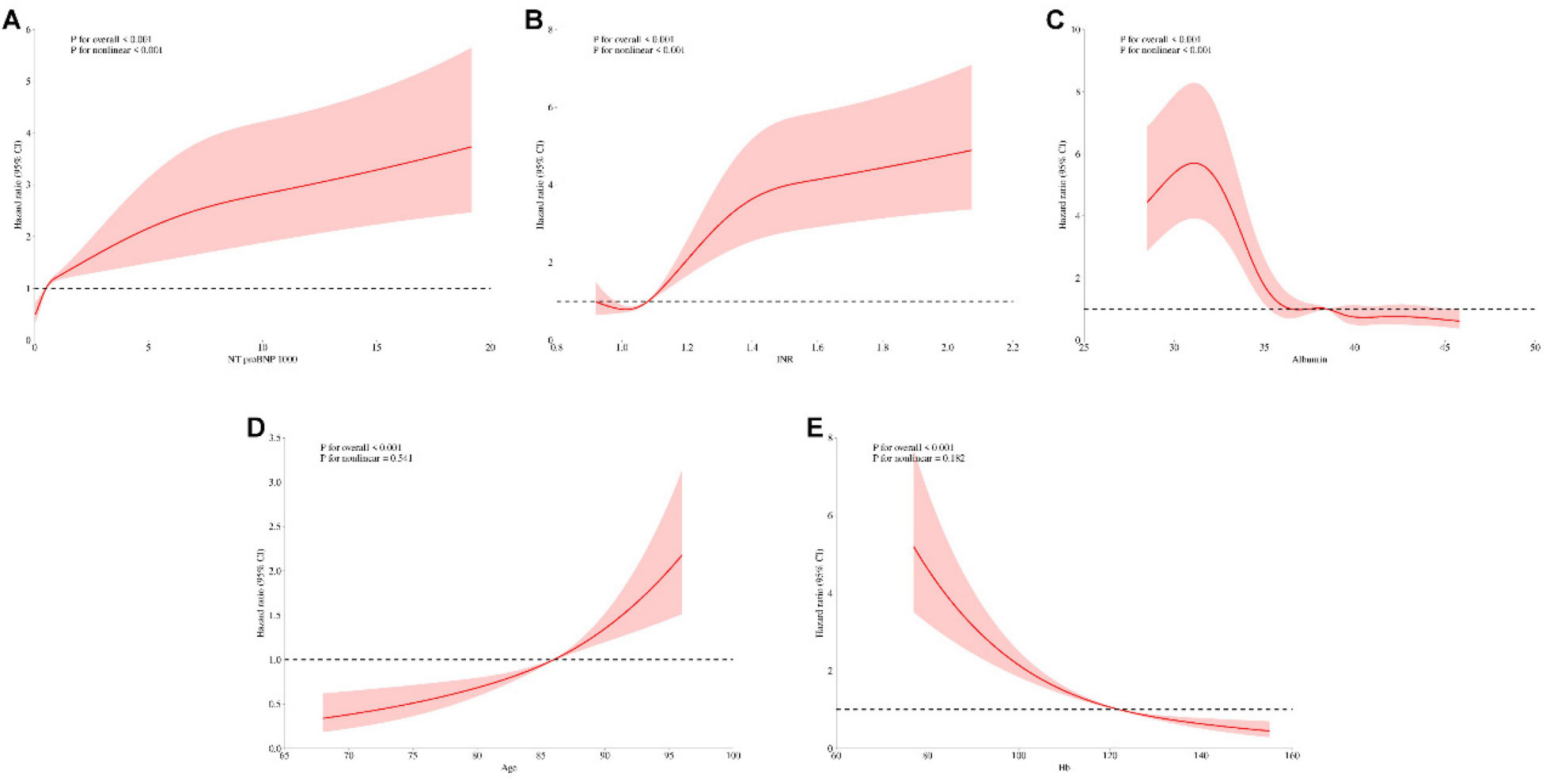
Unadjusted restricted cubic spline analysis of continuous variables and mortality risk. Restricted cubic spline plots for **(A)** NT-proBNP, **(B)** INR, **(C)** albumin, **(D)** age, and **(E)** hemoglobin.

To investigate the nonlinear relationships between continuous predictors and mortality risk, we conducted adjusted analyses of key predictors identified in multivariable Cox regression models, sequentially excluding each variable under investigation (Figure 11). In adjusted analyses, NT-proBNP, albumin, and INR demonstrated significant nonlinear associations with mortality (global *P* < 0.001, nonlinearity *P* < 0.001). Age and hemoglobin exhibited strong linear associations with mortality (global *P* < 0.001) with marginally significant nonlinear components (nonlinearity *P* = 0.541 and 0.182, respectively). These patterns remained consistent across both unadjusted and adjusted analyses, confirming the robustness of these relationships independent of other clinically significant predictors.

## Discussion

In this comprehensive investigation of outcomes in patients with coronary artery disease and diabetes comorbidity, we developed and validated a multimodal prediction model. The model demonstrated robust predictive performance, with notable mortality discrimination accuracy and consistent calibration across various time intervals. The complementary nature of these parameters suggests that multimodal approaches may more effectively capture the complex pathophysiology underlying disease progression in this comorbid population. To facilitate clinical decision-making, we developed a nomogram based on our model, which allows clinicians to estimate a patient's survival probabilities at 3, 5, and 8 years. The nomogram incorporates key predictors such as age, hemoglobin levels, albumin, INR, and NT-proBNP, which are factors that clinicians commonly encounter in daily practice. By assigning a score to each of these variables, the nomogram calculates a total points score that can be translated into predicted survival probabilities at different time intervals. This tool can be integrated into clinical workflows as a decision-support system, assisting clinicians in risk-adjusted treatment planning tailored to individual patients. For instance, when faced with a patient exhibiting specific risk factors (e.g., advanced age or low albumin levels), a clinician can use the nomogram to estimate the patient's long-term survival prognosis, which may influence decisions regarding aggressive interventions, frequency of follow-up visits, or the need for specialized care.

Our study identified anemia as a significant prognostic predictor in patients with comorbid coronary artery disease and diabetes. Anemia, a known contributor to ischemic conditions, reduces blood oxygen capacity, causing myocardial hypoxia. Compensatory mechanisms such as increased heart rate and cardiac output exacerbate pre-existing myocardial ischemia (15). Furthermore, anemia frequently coexists with chronic kidney disease (CKD). Impaired renal erythropoietin (EPO) production in CKD patients significantly elevates anemia risk (16). This creates a pathophysiological cycle where anemia worsens renal hypoxia, accelerating kidney function deterioration. Importantly, renal insufficiency promotes toxin accumulation, intensifying systemic inflammatory responses and oxidative stress that compound cardiovascular damage (17). Current evidence demonstrates no conclusive benefit of anemia correction for improving clinical outcomes in cardiac patients (18). Robust clinical data indicate that erythropoiesis-stimulating agents (ESAs) provide neither symptomatic relief nor outcome improvement in those with mild-to-moderate anemia, while potentially increasing serious adverse risks (19).

Inflammation serves as a key driver in the initiation and progression of atherosclerosis (20). In diabetic patients, inflammatory responses exacerbate vascular endothelial damage, accelerating lipid deposition and plaque formation. Furthermore, these responses activate the coagulation system, elevating clotting factor levels and platelet activity, thereby increasing thrombotic risk. This reciprocal interaction between coagulation and inflammation creates a self-reinforcing cycle that accelerates atherosclerotic progression (21, 22). INR levels reflect underlying coagulation dysfunction and correlate with clinical outcomes. Elevated INR levels demonstrate strong prognostic value, showing consistent associations with adverse outcomes in cardiovascular disease patients (23–25). The conversion of fibrinogen to insoluble fibrin and subsequent formation of stable clots represents the terminal phase of the coagulation cascade. Impaired fibrin clot architecture is widely recognized as both a prognostic marker in CAD patients and a potential indicator of thromboembolic risk (26, 27). Furthermore, hyperfibrinogenemia may constitute a key mechanism underlying elevated cardiovascular risk in T2DM populations (28, 29).

Albumin, the most abundant circulating protein in humans, exhibits critical physiological functions including anti-inflammatory effects, antioxidant activity, and inhibition of platelet aggregation (30). As the predominant antioxidant in whole blood, serum albumin derives its antioxidant capacity primarily from free cysteine residues. The prognostic significance of serum albumin in cardiovascular diseases stems principally from its associations with nutritional status and systemic inflammation (31). Albumin additionally modulates inflammatory and immune responses while maintaining vascular integrity (32). Novel albumin-based inflammatory indices such as the C-reactive protein-albumin-lymphocyte (CALLY) index (33) and C-reactive protein/albumin ratio (CAR) (34) enhance prognostic assessment by quantifying systemic inflammatory burden. Reduced serum albumin levels demonstrate associations with ischemic heart disease, heart failure, atrial fibrillation, stroke, and venous thromboembolism (35). In diabetic patients, albumin's antioxidant properties become compromised, acquiring pro-oxidant characteristics (36). Elena The work of Succurro et al. (37) reveals that reduced albumin in diabetes may upregulate Nox2 activity—a mediator of pro-inflammatory responses and vasoconstriction—potentially contributing to cardiovascular events.

Cardiac contraction primarily relies on energy derived from mitochondrial fatty acid oxidation, a process supported by the abundance of mitochondria in myocardial tissue. NT-proBNP serves as a key prognostic biomarker for risk stratification in heart failure patients (38). Pathological cardiac hypertrophy is characterized by reduced fatty acid oxidation alongside enhanced glucose uptake and glycolytic activity. In T2DM, insulin resistance restricts myocardial glucose utilization for energy production, while increased fatty acid oxidation, lipid accumulation, and oxygen consumption collectively impair cardiac function (39). The Malachias et al. study identified NT-proBNP as a significant predictor of mortality and cardiovascular events in diabetic populations (40). Incorporating both BNP and NT-proBNP into optimized risk prediction models enhances discriminative capacity beyond established cardiovascular outcome predictors (41). Serial NT-proBNP measurements demonstrate superior predictive value over single assessments for forecasting cardiovascular mortality or heart failure hospitalization in patients with coronary artery disease and T2DM (42).

Our study revealed that diuretic and digoxin use in patients with comorbid coronary artery disease and diabetes was associated with poorer clinical outcomes, likely attributable to their more complex comorbidities and disease progression toward heart failure. However, prior research has demonstrated that loop diuretic use in coronary artery disease patients without systolic heart failure or renal impairment correlates with increased cardiovascular and non-cardiovascular mortality (43). These findings emphasize the necessity for judicious prescription of these medications in patients lacking clear clinical indications.

Given the multifactorial, multisystem complexity of coronary artery disease (CAD) with comorbid diabetes, there is an urgent clinical need for precise prognostic models to guide therapeutic decision-making. Machine learning demonstrates significant potential in risk prediction, early diagnosis, and personalized management of CAD with diabetes. By analyzing extensive clinical datasets, these algorithms can identify complex patterns potentially overlooked by conventional statistical methods, offering novel insights for early intervention (44–46). Our investigation employed a comprehensive multimodal approach with rigorous validation protocols and clinically relevant time frames. The model demonstrated robust discriminative performance and calibration accuracy across temporal intervals, suggesting practical utility for both short- and long-term risk stratification. However, limitations include the single-center study design with a restricted sample size (*n* = 389), which may limit generalizability, as well as the lack of external validation using multi-ethnic data. Future validation in larger, multicenter cohorts is required to confirm clinical applicability**.** Given the relatively small sample size of 389 patients and the inclusion of multiple predictors, the risk of overfitting is a concern, especially in a longitudinal study with potential patient loss to follow-up. To mitigate this, we employed Random Survival Forest (RSF), which reduces overfitting through ensemble learning, enhances model generalizability, and allows for the interpretability of predictor contributions. Another limitation is that we did not implement a formal time-dependent Cox model; medication exposures were aggregated over the first six months and treated as baseline covariates, so residual immortal time bias may remain. In addition to the methods used in this study, we acknowledge the potential benefits of exploring auxiliary model-based optimization methods and hybrid nonlinear identification algorithms, such as those discussed by Ali et al. (47, 48), which could offer further improvements in predictive accuracy. These advanced techniques are particularly beneficial for handling complex, non-linear relationships in clinical data, and could enhance the model's robustness and adaptability to diverse patient populations (49, 50). Specifically, the **Runge-Kutta optimization algorithm** for fractional input nonlinear output error systems, as well as **Chameleon Swarm Optimization** and **Mountain Gazelle optimization algorithms**, offer biologically inspired, auxiliary model-driven frameworks that can improve both interpretability and dynamic response estimation of medical survival models, enabling more precise and reliable predictions in clinical settings. We are actively considering these approaches for future iterations of our model to improve its performance in more dynamic clinical settings.

## Conclusion

The advancement of machine learning has established random forest models as a robust methodology for developing medical prediction tools. Integration of refined biomarkers with artificial intelligence-driven models shows significant potential to enhance prognostic assessment in patients with coronary artery disease and comorbid diabetes. This study conducted a comprehensive analysis of clinical parameters associated with mortality risk in coronary artery disease patients with diabetes comorbidity. Our findings highlight the critical predictive value of hemoglobin, INR, albumin, NT-proBNP, age, fibrinogen, diuretic use, and digitalis therapy in risk stratification. The developed model demonstrates reliable short-term prognostic accuracy using a parsimonious set of clinical variables. This advancement holds promise for enabling personalized therapeutic strategies to optimize clinical outcomes in this high-risk population.

## Data Availability

The raw data supporting the conclusions of this article will be made available by the authors, without undue reservation.
