# Supplementary material for: Explainable machine learning model of coronary artery disease combined with diabetes: development and validation study
Source: Front Cardiovasc Med. 2026 Jan 5;12:1674287. doi: 10.3389/fcvm.2025.1674287 (PMC12812605; doi:10.3389/fcvm.2025.1674287)
Supplement: Supplementary file 1 [file Datasheet1.docx]

**Supplementary Table 1** **Detailed Clinical and Characteristics of the Total Study Population**

|  | [ALL] N=389 | N |
| --- | --- | --- |
| Death |  | 389 |
| No | 90 (23.14%) |  |
| Yes | 299 (76.86%) |  |
| Survival time(year) | 4.68 [0.55;8.71] | 389 |
| Gender | 86.00 [82.00;90.00] | 389 |
| Female | 24.46 [22.32;26.75] | 389 |
| Male | 72.00 [65.00;80.00] | 389 |
| Age | 135.40 (13.41) | 389 |
| BMI (kg/m²) | 68.60 [62.80;73.60] | 389 |
| HR (bpm) | 4.68 [0.55;8.71] | 389 |
| mSBP (mmHg) | 86.00 [82.00;90.00] | 389 |
| mDBP (mmHg) | 24.46 [22.32;26.75] | 389 |
| Smoke |  | 389 |
| Never/ Former smoker | 243 (62.47%) |  |
| Current smoker | 146 (37.53%) |  |
| Hypertension |  | 389 |
| No | 48 (12.34%) |  |
| Yes | 341 (87.66%) |  |
| CHD |  | 389 |
| No | 268 (68.89%) |  |
| Yes | 121 (31.11%) |  |
| CHF |  | 389 |
| No | 262 (67.35%) |  |
| Yes | 127 (32.65%) |  |
| CKD |  | 389 |
| No | 317 (81.49%) |  |
| Yes | 72 (18.51%) |  |
| AF |  | 389 |
| No | 217 (55.78%) |  |
| Yes | 172 (44.22%) |  |
| Anemia |  | 389 |
| No | 243 (62.47%) |  |
| Yes | 146 (37.53%) |  |
| Glucose | 5.80 [4.98;7.18] | 389 |
| NT_proBNP_1000(pg/mL/1000) | 0.50 [0.18;1.91] | 389 |
| Hb (g/L) | 122.00 [107.00;134.00] | 389 |
| TC (mmol/L) | 3.65 [3.16;4.28] | 389 |
| TG (mmol/L) | 1.32 [0.99;1.92] | 389 |
| HDL (mmol/L) | 1.01 [0.84;1.19] | 389 |
| LDL (mmol/L) | 2.03 [1.54;2.52] | 389 |
| Albumin (g/L) | 38.50 [35.00;40.60] | 389 |
| Scr (μmol/L) | 90.80 [72.00;123.00] | 389 |
| UA (μmol/L) | 330.50 [237.60;420.00] | 389 |
| Na (mmol/L) | 139.00 [136.00;141.00] | 389 |
| K (mmol/L) | 4.00 [3.78;4.31] | 389 |
| Ca (mmol/L) | 2.23 [2.13;2.32] | 389 |
| P (mmol/L) | 1.10 [0.96;1.25] | 389 |
| NLratio | 2.48 [1.80;4.09] | 389 |
| Fibrinogen (g/L) | 3.49 [2.93;4.22] | 389 |
| INR | 1.08 [1.01;1.15] | 389 |
| CRP (mg/L) | 0.68 [0.18;2.73] | 389 |
| eGFR (mL/min/1.73m²) | 67.35 (24.40) | 389 |
| IVS (mm) | 11.00 [10.00;12.00] | 389 |
| LVPW (mm) | 10.00 [10.00;11.00] | 389 |
| LVESD (mm) | 33.00 [31.00;35.00] | 389 |
| LVEDD (mm) | 49.00 [47.00;51.00] | 389 |
| LVEF (%) | 59.00 [55.00;62.00] | 389 |
| LVMI (g/m2) | 32.00 [29.00;34.00] | 389 |
| Aspirin |  | 389 |
| No | 208 (53.47%) |  |
| Yes | 181 (46.53%) |  |
| Clopidogrel |  | 389 |
| No | 144 (37.02%) |  |
| Yes | 245 (62.98%) |  |
| β-blocker |  | 389 |
| No | 106 (27.25%) |  |
| Yes | 283 (72.75%) |  |
| CCB |  | 389 |
| No | 111 (28.53%) |  |
| Yes | 278 (71.47%) |  |
| Nitrate |  | 389 |
| No | 50 (12.85%) |  |
| Yes | 339 (87.15%) |  |
| ACEI/ARB |  | 389 |
| No | 174 (44.73%) |  |
| Yes | 215 (55.27%) |  |
| Statins |  | 389 |
| No | 129 (33.16%) |  |
| Yes | 260 (66.84%) |  |
| Diuretics |  | 389 |
| No | 151 (38.82%) |  |
| Yes | 238 (61.18%) |  |
| Digitalis |  | 389 |
| No | 318 (81.75%) |  |
| Yes | 71 (18.25%) |  |

**Supplementary Table 2. Variables Selected by LASSO Regression with Their Coefficients**

| **Variable** | **Coefficient** |
| --- | --- |
| Age | 0.040 |
| HR | 0.000 |
| mDBP | -0.003 |
| Hb | -0.011 |
| TG | 0.014 |
| HDL | -0.120 |
| Albumin | -0.035 |
| K | 0.153 |
| P | 0.308 |
| Fibrinogen | 0.100 |
| INR | 0.212 |
| CRP | 0.025 |
| IVS | 0.031 |
| LVEF | -0.002 |
| LVMI | -0.011 |
| AF | 0.041 |
| Anemia | 0.028 |
| Aspirin | -0.026 |
| ACE/ARB | -0.030 |
| Statins | -0.132 |
| Diuretics | 0.40 |
| Digitalis | 0.154 |
| NT_proBNP_1000 | 0.018 |

**Supplementary Table 3. Variance Inflation Factors of Variables in the Final Multivariate Cox Model**

| **Variable** | **VIF** |
| --- | --- |
| Age | 1.137 |
| Hb | 1.298 |
| Albumin | 1.455 |
| Fibrinogen | 1.283 |
| INR | 1.170 |
| Diuretics | 1.178 |
| Digitalis | 1.336 |
| NT-proBNP/1000 | 1.544 |

**Supplementary Table 4. Optimal Cutoff Values for Continuous Variables in the Survival Analysis**

| **Variable** | **Cutoff Value** | **Statistic** |
| --- | --- | --- |
| Hb | 116 g/L | 6.525 |
| Fibrinogen | 3.72 g/L | 5.334 |
| Age | 85 years | 5.040 |
| INR | 1.12 | 5.267 |
| NT-proBNP | 487.2pg/mL | 6.349 |

**Supplementary Table5 :** **Random Survival Forest Hyperparameter Settings**

| **Hyperparameter** | **Value** |
| --- | --- |
| Number of trees | 1000 |
| Optimal node size | 5 (determined by resampling and minimizing out-of-bag error) |
| Number of variables at each split | 3 |
| Splitting rule | Logrank *random* |
| Resampling method | Simple random sampling without replacement (swor) |
| Resample size | 172 |
| Performance error (OOB CRPS) | 0.2626 |
| Standardized CRPS (OOB) | 0.1554 |
| Number of random split points | 10 |

**Supplementary Table 6: Bootstrap Optimism Correction Results**

| **Cohort** | **Original C-Index** | **Bias** | **Standard Error** | **95% CI (Normal)** | **95% CI (BCa)** |
| --- | --- | --- | --- | --- | --- |
| Training Set | 0.7516 | 0.0030 | 0.0152 | (0.7190, 0.7754) | (0.7190, 0.7749) |
| Validation Set | 0.7597 | 0.0163 | 0.0226 | (0.6984, 0.7890) | (0.7006, 0.7887) |

**Supplementary Table7 : Relevant functions and their corresponding R packages used in this study**

| **Analysis method** | **Main R packages** | **Key purpose / functions** |
| --- | --- | --- |
| **Cox proportional hazards model** | **survival**; **rms** | Provides Surv objects, Kaplan–Meier curves and Cox models; extended modelling and validation. |
| **Random survival forest (RSF)** | **randomForestSRC** | Parallel random forests for survival/competing risks and variable importance. |
| **Time‑dependent ROC curve** | **timeROC**; **riskRegression** | Estimates time‑dependent ROC curves & AUC for censored data; computes time‑dependent AUC and performance measures. |
| **Calibration of survival models** | **rms**; **pec** | Bootstrap/cross‑validation calibration curves for Cox/other survival models; calibration & validation of survival predictions. |
| **Decision curve analysis (DCA)** | **dcurves**; **rmda** | Decision curve analysis & net benefit for binary/time‑to‑event endpoints. |


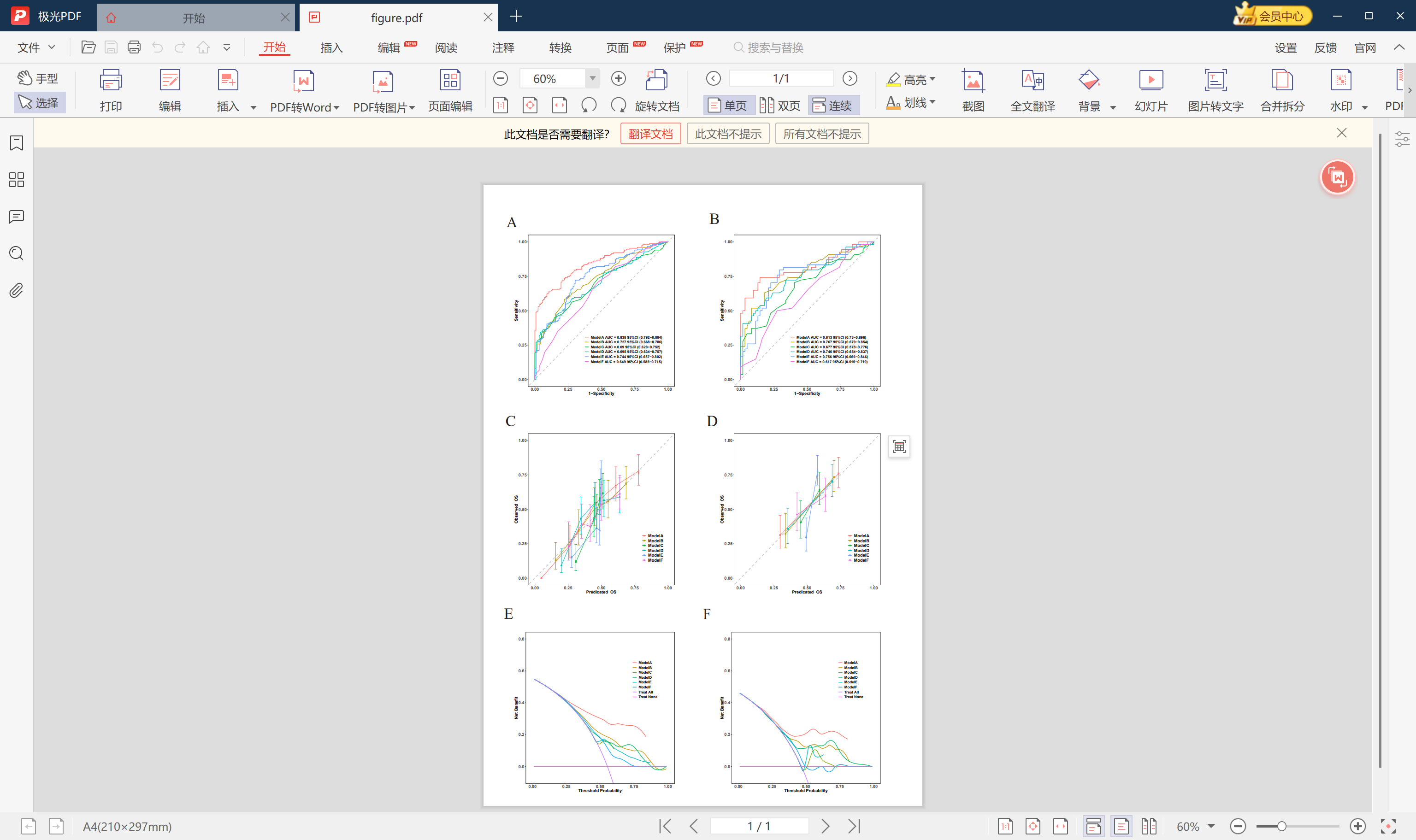


Model A: age + hemoglobin + albumin + fibrinogen + INR + diuretics + digoxin + NT-ProBNP/1000, Model B: hemoglobin, Model C: INR, Model D: albumin, Model E: NT-ProBNP/1000, and Model F：age.

**Supplementary Figure 1. Discriminative Performance of Different Models for 5-Year Mortality Prediction.**


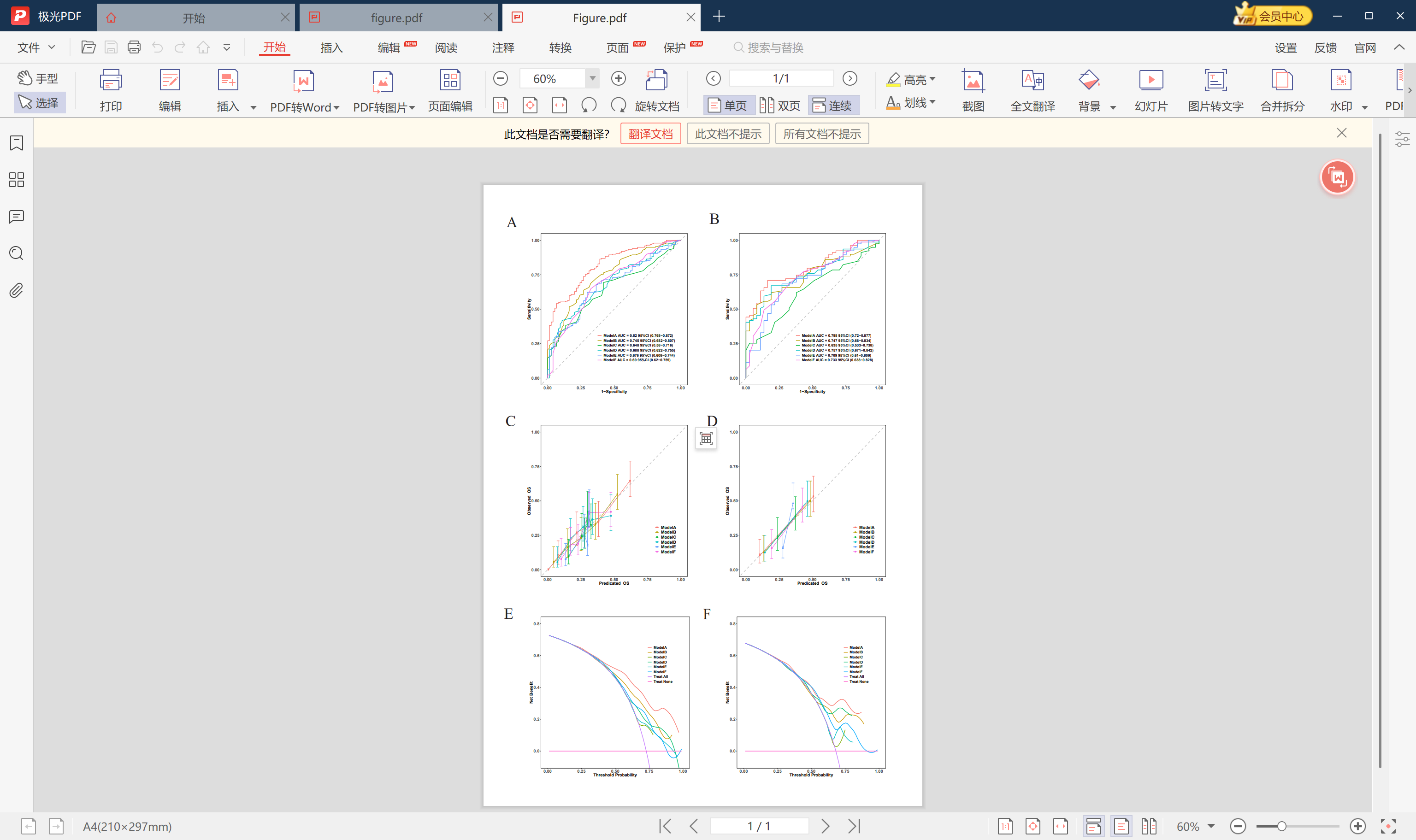


Model A: age + hemoglobin + albumin + fibrinogen + INR + diuretics + digoxin + NT-ProBNP/1000, Model B: hemoglobin, Model C: INR, Model D: albumin, Model E: NT-ProBNP/1000, and Model F：age.

**Supplementary Figure2. Discriminative Performance of Different Models for 8-Year Mortality Prediction.**


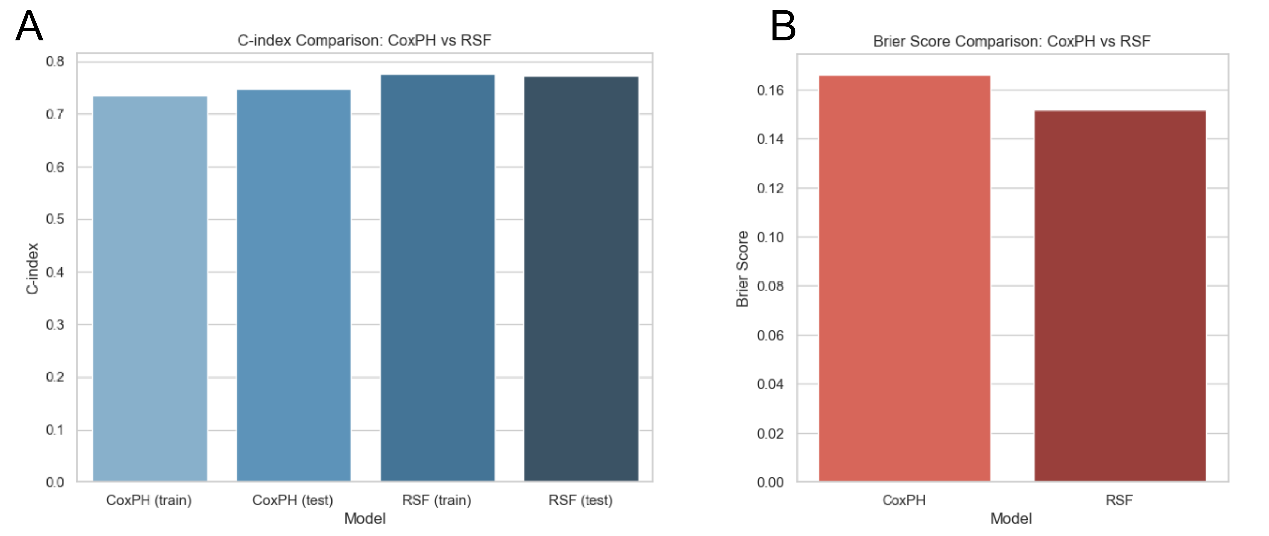


**Supplementary Figure 3: (A) Bar plots of C-index for Cox proportional hazards (CoxPH) and random survival forest (RSF) models in the training and test sets; (B) Bar plots of Brier scores for CoxPH and RSF models**


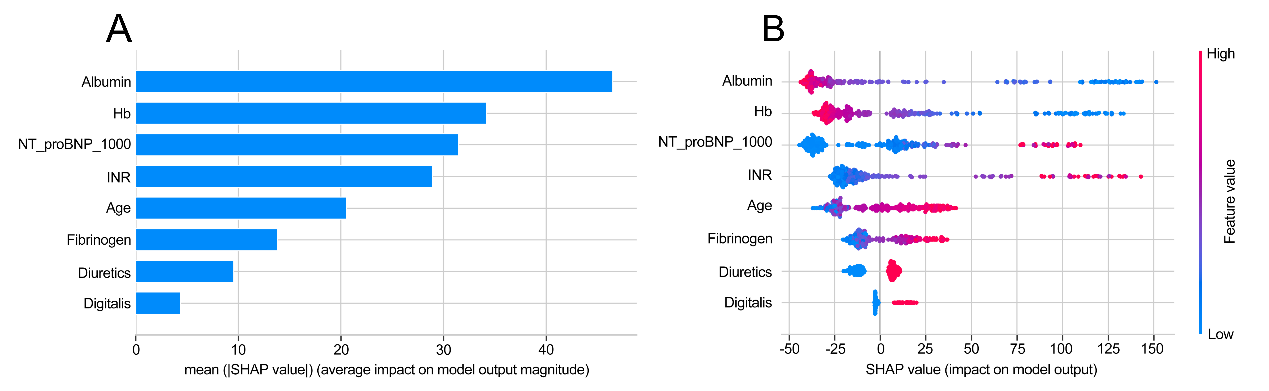


**Supplementary Figure 4: SHAP-based bar plots and beeswarm plots for the random survival forest (RSF) model**


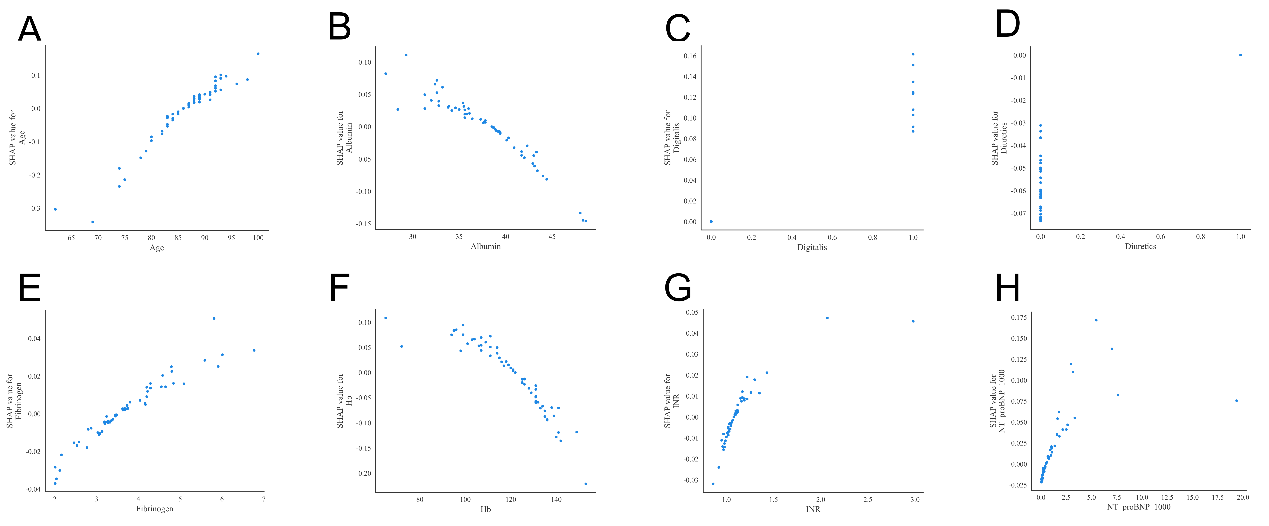


**Figure S5: Partial dependence plots illustrating the relationships between individual variables and the outcome for the random survival forest (RSF) model**


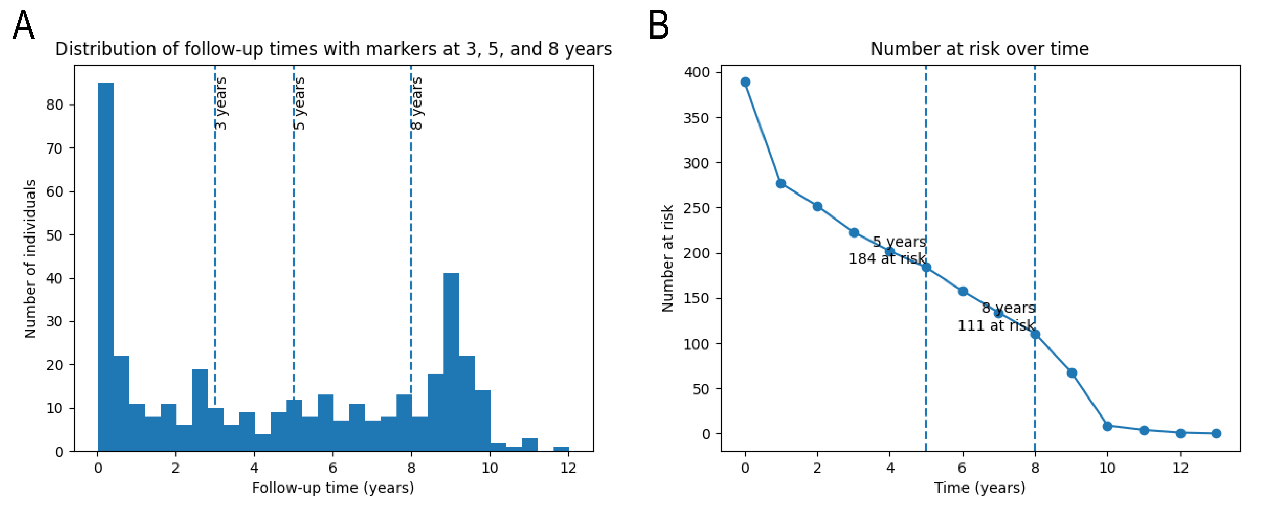


**Figure S6:Plot of Follow-up Distribution and Number of Participants at Risk in This Study**
